# Nitrosative and oxidative stress responses in fungal pathogenicity

**DOI:** 10.1016/j.mib.2009.06.007

**Published:** 2009-08

**Authors:** Alistair JP Brown, Ken Haynes, Janet Quinn

**Affiliations:** 1 Aberdeen Fungal Group, School of Medical Sciences, University of Aberdeen, Institute of Medical Sciences, Foresterhill, Aberdeen AB25 2ZD, UK; 2 Department of Microbiology, Imperial College London, The Flowers Building, London SW7 2AZ, UK; 3 Institute for Cell and Molecular Biosciences, Newcastle University, Newcastle upon Tyne NE2 4HH, UK

## Abstract

Fungal pathogenicity has arisen in polyphyletic manner during evolution, yielding fungal pathogens with diverse infection strategies and with differing degrees of evolutionary adaptation to their human host. Not surprisingly, these fungal pathogens display differing degrees of resistance to the reactive oxygen and nitrogen species used by human cells to counteract infection. Furthermore, whilst evolutionarily conserved regulators, such as Hog1, are central to such stress responses in many fungal pathogens, species-specific differences in their roles and regulation abound. In contrast, there is a high degree of commonality in the cellular responses to reactive oxygen and nitrogen species evoked in evolutionarily divergent fungal pathogens.

## Introduction

The fungal kingdom is remarkably diverse, boasting an estimated 1.5 million species, but only about 400 fungal species are pathogenic to humans. Most invasive mycoses are caused by a small set of fungi that include *Candida*, *Cryptococcus* and *Pneumocystis* species, *Aspergillus fumigatus*, *Histoplasma capsulatum*, *Coccidioides immitis* and *Paracoccidioides brasiliensis*. However, most research on nitrosative and oxidative stress responses has been limited to *Candida* species, *Cryptococcus neoformans* and *A. fumigatus*. Hence this review focuses primarily on these species.

Macrophages, neutrophils and other phagocytic cells generate potent reactive oxygen and nitrogen species (ROS and RNS), which are toxic to most fungal pathogens, causing damage to DNA, proteins and lipids [1]. However, fungal pathogens mount robust responses that detoxify these chemicals and repair the molecular damage they cause. These oxidative and nitrosative stress responses help fungal pathogens to survive their initial contacts with the host immune system and are crucial for disease establishment [2].

Fungal pathogens display differing degrees of evolutionary adaptation to their human host and hence to host immune defences. At one extreme, *A. fumigatus* is an abundant saprophyte that is associated mainly with decaying vegetation. However, it can cause devastating infections in immunocompromised patients because of its natural abundance, its ability to colonize the alveoli, and its efficacy in adapting to hostile environments. In contrast, *Pneumocystis jiroveci*, is evolutionarily adapted to the alveolar epithelium, and appears unable to grow *ex vivo* having shed normally essential metabolic functions during its co-evolution with its host. Whether evolutionarily well adapted to humans or not, fungal pathogens have become relatively resistant to the chemical insults they receive from their host [3].

Fungal pathogens display different routes of infection and this influences their exposure to the chemical armoury of the host. For example, *C. neoformans*, *H. capsulatum* and *A. fumigatus,* are generally acquired by inhalation. In contrast, *Candida albicans* has evolved as a commensal that survives in the gastrointestinal and/or urogenital tracts of healthy individuals. Therefore, differing infection routes may also have contributed to the evolutionary divergence of stress responses in these species.

This brief review summarizes our current understanding of nitrosative and oxidative stress responses in fungal pathogens, highlighting recent advances in the field.

## The immune cell armoury

**Figure 1:**
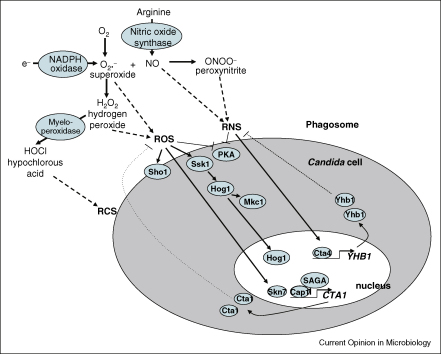
Generation of ROS and RNS in the phagosome, and the proteins and pathways involved in oxidative and nitrosative stress responses in *C. albicans*.

As illustrated in Figure 1, phagocytic cells synthesize an array of toxic chemicals that promote fungal killing (reviewed in [1,4]). ROS such as superoxide (O_2_^−^) ions are generated by the partial reduction of oxygen during aerobic respiration in the mitochondrion. However, following cytokine activation, phagocytic cells synthesize large amounts of ROS and RNS. NADPH oxidase becomes activated leading to the generation of O_2_^−^, which can be converted to hydrogen peroxide (H_2_O_2_) by superoxide dismutase, or to hydroxyl anions (OH^−^) and hydroxyl radicals (•OH) via the Haber–Weiss reaction. The resultant H_2_O_2_ can also generate hypochlorous acid (HOCl) by myeloperoxidase. In phagocytic cells, nitric oxide synthase (NOS2) is induced in response to cytokine stimulation and other immunological stimuli. This nitric oxide synthase generates a range of RNS including the NO radical and nitrite (NO_2_^−^). NO reacts with superoxide to form peroxynitrite (ONOO^−^), and the combination of nitrite and hypochlorous acid yields nitryl chloride (NO_2_Cl). Therefore, phagocytic cells are capable of generating a toxic cocktail of reactive oxygen, nitrogen and chlorine species that can oxidize, nitrosylate and chlorinate amino acids, nucleotides and/or lipids.

ROS and RNS contribute to the killing of fungal pathogens such as *A. fumigatus*, *C. albicans* and *C. neoformans*, by host immune cells [1,2,5]. This is reflected in the ability of exogenous ROS and RNS to block the growth or kill these fungi *in vitro* [6^•^,7,8]*.* Indeed, ROS induce programmed cell death in *C. albicans* [9]. However, the effectiveness of a particular host defence mechanism in killing a fungal pathogen depends upon the susceptibility of that pathogen to ROS and RNS. Mice with defects in phagocytic superoxide production are more susceptible to *A. fumigatus* and *C. albicans* [10]. However, inactivating the inducible nitric oxide synthase (NOS2) does not make mice more susceptible to *C. albicans* infection, suggesting that NO production might not be the main defence against *C. albicans* in such infections [11]. Nevertheless, RNS are protective in oral candidiasis [12]. Also, the interferon-γ induced anti-cryptococcal activity of murine macrophages depends more on RNS than ROS [13], whereas the killing of *A. fumigatus* by murine macrophages is more dependent upon ROS [14]. Therefore, different weapons in the fungicidal chemical armoury have different degrees of potency against particular pathogens.

The chemical armoury is generally activated by immune cells when they come into contact with fungal pathogens [2]. For example, *C. albicans* and *C. neoformans* activate an oxidative burst in macrophages [2,5], and *A. fumigatus* also stimulates nitric oxide production in alveolar macrophages [15]. However, this activation can depend upon the experimental context. For example, *C. neoformans* stimulates RNS synthesis during cryptococcal meningoencephalitis in mice, and following contact with rat alveolar macrophages, but not in cultured mouse macrophages [15–17]. This caveat should be borne in mind when interpreting experimental data.

## The responses of fungal pathogens

Studies in the model yeasts *Saccharomyces cerevisiae* and *Schizosaccharomyces pombe* have provided a framework for the study of the oxidative stress response in pathogenic fungi. However, it is now clear that these benign yeasts differ from fungal pathogens in many aspects of oxidative stress regulation. Furthermore, studies in fungal pathogens *per se* have resulted in recent advances in our understanding of nitrosative stress responses.

The array of stress regulators identified thus far as being required for wild-type levels of oxidative or nitrosative stress resistance in the major pathogenic fungi are summarized in Table 1, and the cellular organization of these proteins in *C. albicans* is illustrated in Figure 1. It is apparent that mitogen activated protein kinase (MAPK) pathways play a central role in oxidative stress responses in many fungal pathogens [18,19,20^•^,21]. The Hog1 stress activated protein kinase (SAPK) is robustly activated by H_2_O_2_ in *C. albicans* cells [18,19]. However, Hog1 does not play a major role in the transcriptional response to ROS in *C. albicans* [22], and its exact function in combating oxidative stress remains unknown. Recent evidence suggests that Hog1 inactivation affects respiratory function [23], although it is unclear whether this underlies the sensitivity of *hog1* cells to ROS. It is known, however, that two-component signalling is important for the relay of oxidative stress signals to the SAPK module in both *C. albicans* and *C. neoformans* as the response regulator protein Ssk1 is required for peroxide induced activation of Hog1 [24,25]. Whilst the *C. albicans* peroxide sensing histidine kinase(s) remains elusive, in *C. neoformans* the Tco2 histidine kinase is partially responsible for the sensing and relay of the peroxide signal to Ssk1 [25]. Interestingly, studies on SAPK signalling in *C. neoformans* uncovered a strikingly different pattern of Hog1 phosphorylation in the most virulent serotype A cells, compared to less virulent serotypes [20^•^]. In particular, extremely high basal levels of Hog1 phosphorylation are seen in serotype A cells, which arise as a result of sequence differences in the upstream protein kinase Ssk2 [26^•^], and this results in increased resistance to ROS and other stresses.

Intriguingly, the function of the transmembrane protein Sho1, which in *S. cerevisiae* relays osmotic stress signals to the Hog1 SAPK, appears to have been reassigned to oxidative stress signalling in pathogens such as *C. albicans* [27] and *A. fumigatus* [28]. It is not clear how this signalling is mediated, but in *C. albicans* it is independent of the Hog1 pathway [27].

In addition to Hog1, the cell wall integrity MAPK is also activated by stress in *C. albicans* and *C. neoformans* [29^•^,30^•^]. In *C. neoformans*, the Mpk1 MAPK responds both to peroxide and nitrosative stress in a protein kinase C (Pkc1) dependent mechanism [30^•^]. Interestingly, whilst *pkc1*Δ cells are sensitive to both oxidative and nitrosative stress, *mpk1*Δ cells are not, indicating that Pkc1 must regulate additional pathways in response to these stresses. Significantly, this study is the first report that Pkc1 is important for the response to nitrosative stress in fungi. In *C. albicans* the analogous MAPK, Mkc1, is also activated in response to oxidative stress. Intriguingly, in this fungus, ROS-induced activation of Mkc1 is dependent on the Hog1 SAPK [29^•^]. As seen in *C. neoformans*, *C. albicans* cells lacking the Mkc1 MAPK do not display increased sensitivity to ROS. Thus in *C. albicans*, Hog1 regulation of the oxidative stress response must involve targets in addition to Mkc1.

In contrast to the positive regulators described above, the cAMP signalling pathway negatively regulates both oxidative and nitrosative stress responses in *C. albicans*. For example, induction of the pathway by inactivation of the phosphodiesterase Pde2, which degrades cAMP, results in increased sensitivity to both ROS and RNS [31,32]. Whilst, the negative regulation of key oxidative stress regulators by cAMP signalling has previously been shown in *S. cerevisiae*, the study by Bahn *et al*. [31] is the first report linking downregulation of the cAMP/protein kinase A pathway with resistance to nitrosative stress.

Regarding transcriptional regulators of the oxidative stress response, orthologues of important factors in *S. cerevisiae* such as the AP-1 like factor Yap1 and the Skn7 response regulator transcription factor have been characterized in several pathogenic fungi (Table 1). In *S. cerevisiae*, Yap1 collaborates with Skn7 to regulate many oxidative stress-response genes and a recent study indicates that this is also the case in *C. glabrata*, as single or double *C. glabrata yap1* *skn7* mutants are equally sensitive to H_2_O_2_ [33^•^]. In *C. albicans* the AP1-like transcription factor Cap1 is the major regulator of the oxidative stress-induced transcriptome and proteome both *in vitro* [34,35] and *ex vivo*, following exposure to neutrophils [36^•^]. Recent work, employing Chip on chip experiments, detected Cap1 binding to 89 target genes [37]. Notably however, Cap1 binding was not restricted to promoters of these target genes [37]. Furthermore, an important function of Cap1 is to recruit the SAGA/ADA co-activator complex, which regulates histone acetylation, to the promoters of oxidative stress responsive genes [38]. As observed in model yeasts, *C. albicans* Cap1 and *A. fumigatus* Yap1 accumulate in the nucleus in response to H_2_O_2_ [39,40]. In *S. cerevisiae,* Yap1 is activated by oxidation of specific cysteine residues present in two cysteine-rich domains (CRDs) that prevents the interaction with the Crm1 nuclear export factor. The same basic mechanism is conserved in *S. pombe*, although intriguingly, the mechanisms underlying oxidation of the AP-1 like transcription factors have diverged in these model yeasts. In *C. albicans,* mutation of the carboxy-terminal CRD affects Cap1 regulation [39], suggesting that the basic mechanism of oxidation is conserved in this pathogen. Whilst the precise mechanism underlying Cap1 regulation is unknown, it has been established that, unlike *S. pombe* Pap1, Cap1 function is independent of the Hog1 SAPK [17,18]. Interestingly, *C. neoformans* does not contain a well conserved Yap1 homologue. Instead, an ATF/CREB-like gene *ATF1* is needed for the induction of *TRX1* in response to oxidative stress, and an *atf1*Δ mutant is sensitive to oxidative stress [41^•^].

Similar to that seen in *S. cerevisiae*, the Cys_2_His_2_ zinc finger transcription factors Msn2 and Msn4 mediate a core stress response in *C. glabrata* [42]. A separate study revealed that Msn4 functions in parallel with Yap1 and Skn7 to mediate oxidative stress resistance [33^•^]. However, the function of Msn2/4-like proteins has diverged significantly in *C. albicans* where they have no detectable role in mediating responses to ROS or indeed a core stress response [43]. Interestingly, the HD1 (homology domain 1) motif of Msn2/4, which is important for stress-regulated intracellular localization, is only present in close relatives of *S. cerevisiae* such as *C. glabrata* and not, for example, in *C. albicans* [42].

Less is known about transcriptional regulators of the nitrosative stress response. Interestingly however, recent work revealed that in *C. albicans,* RNS induced gene expression is primarily regulated by the Zn(II)_2_-Cys_6_ transcription factor, Cta4 [44^••^]. A nitric oxide-responsive element in the promoter of the *C. albicans* flavohaemoglobin *YHB1* gene was identified. Magnetic beads coated with this regulatory DNA sequence were used to isolate putative transcriptional regulators from *C. albicans* extracts. This elegant approach led to the identification of Cta4 as a major regulator of *YHB1* induction and resistance to nitrosative stress in this pathogen [44^••^]. In contrast, the analogous response in *S. cerevisiae* is regulated by the Cys_2_His_2_ zinc finger transcription factor, Fzf1. The only other information regarding regulators of RNS induced gene expression has come from a study in *C. neoformans*, where the AP-1 like factor Yap4 was shown to regulate the expression of *TRX1* in response to nitrosative stress. However, Yap4 is not thought to be the major regulator of RNS-stimulated gene expression in *C. neoformans* [41^•^]. Clearly more remains to be learned regarding the signalling and transcriptional networks that regulate the response to RNS in fungal pathogens.

Despite divergence in stress signalling pathways, fungal pathogens appear to activate analogous sets of oxidative and nitrosative stress genes to benign model yeasts. For example, analyses of the oxidative stress induced transcriptome and proteome showed that *C. albicans* responds to ROS by activating the synthesis of detoxification mechanisms that include catalase, superoxide dismutase and components of the thioredoxin and glutaredoxin systems [22,35,45]. Similar responses to peroxide treatment have also been reported in *C. glabrata* [42]. In *C. albicans*, the key oxidative stress responsive transcription factor gene *CAP1*, is also induced by peroxide [35], and, as seen in other fungi, the responses evoked in *C. albicans* depend upon the dose of peroxide [26^•^,45].

Divergent fungal pathogens also display common features in their genome-wide responses to nitrosative stress. The response of *C. neoformans* to RNS stress has been examined both by transcript profiling and proteomics [46^•^]. Notably, functions involved in the repair of damage caused by the stress (e.g. chaperones, oxidoreductase and thioredoxin reductase) and the detoxification of the stress (e.g. flavohaemoglobin denitrosylase and NADPH dehydrogenase) are induced [46^•^]. The *H. capsulatum* transcriptome responds in a similar fashion, whereby genes involved in protein refolding and degradation (chaperones, polyubiquitin) and detoxification (nitric oxide reductase, arginase, catalase) are induced [47^•^]. Similarly, *C. albicans* activates genes involved in the RNS detoxification and repair (e.g. nitric oxide dioxygenase, catalase and flavohaemoglobin) [11], at least some of which are under the control of the transcription factor Cta4 [44^••^]. A further common response to RNS exhibited by these evolutionarily divergent fungal pathogens is the induction of genes involved in iron acquisition. Many stress protective enzymes require iron as a cofactor.

Although these global responses to ROS and RNS have been defined *in vitro*, they are relevant to the infection process. Key signatures of these *in vitro* responses are reflected in the genome-wide responses of fungal pathogens to their host, for example, following phagocytosis by macrophages or neutrophils [48–50]. Thioredoxin genes are upregulated in *C. neoformans* during cryptococcal meningitis [51], and oxidative and nitrosative stress genes are upregulated in *C. albicans* following exposure to human blood or mucosal tissue [36^•^,52]. However, these responses are less apparent in the *C. albicans* transcriptome during peritoneal infections [53]. This correlates well with an analysis of oxidative stress gene expression in different experimental infection models using diagnostic GFP fusions [54^•^]. This study revealed that the oxidative stress response is activated after phagocytosis by neutrophils, but not in most *C. albicans* cells infecting the kidney. This suggests that adaptation to oxidative stress might be crucial in the early stages of systemic *C. albicans* infections, but less important once these infections are established.

Pathogenic *Candida* species, and *C. glabrata* in particular, are relatively resistant to oxidative stress [3,33^•^]. Furthermore, stationary phase *C. albicans* and *C. glabrata* cells are considerably more resistant to ROS than exponential cells [18,33^•^]. This is consistent with studies in benign model yeasts, where stress resistance is generally associated with a decrease in growth rate (reviewed in [55]). This suggests that, from the perspective of the fungal pathogen, it might be vital to balance optimal cell growth against stress resistance to proliferate in the host. Thus it will be interesting to examine the relationship between growth and stress gene relation in fungal pathogens.

The analysis of stress regulatory mutants has reinforced the view that robust ROS and RNS responses contribute to fungal pathogenicity (Table 1). For example, *C. albicans* cells lacking the Hog1 SAPK display attenuated virulence [56]. Furthermore, the inactivation of ROS detoxification enzymes has been shown to attenuate the virulence of both *C. albicans* and *C. neoformans* (e.g. see [41^•^,57]). Indeed, the inactivation of cell surface superoxide dismutases was recently proposed to provide a mechanism whereby *C. albicans* can escape host immune surveillance through *ROS* detoxification [58]. In addition, the putative flavohaemoglobin (Yhb1), which is involved in RNS detoxification, is also important for virulence [6^•^,11]. The virulence of *C. neoformans* is further attenuated when the *yhb1* mutation is combined with either S-nitrosoglutathione reductase or superoxide dismutase mutations [6^•^]. Furthermore, the inactivation of mycelial catalases in *A. fumigatus* delays aspergillosis in the rat model of infection, suggesting that they transiently protect this pathogen from the host [59]. Therefore, robust ROS and RNS stress responses generally promote the virulence of fungal pathogens. However, there are some exceptions to this. For example, Cuéllar-Cruz *et al*. have reported that whilst the deletion of the catalase gene, *CTA1*, makes *C. glabrata* cells more sensitive to ROS, this does not attenuate the virulence of this pathogen [33^•^]. This observation might reflect the high doses of *C. glabrata* used in their mouse model of systemic infection, their use of immunosuppressed mice [33^•^], the apparent lack of activation of the oxidative stress response during some tissue infections in mice [54^•^] or the differential dependence of fungal pathogens upon ROS and RNS responses. Furthermore, as described above, the activation of host ROS and RNS depends upon the experimental context, with *C. neoformans* stimulating RNS synthesis under some circumstances, but not others [15–17]. Thus we conclude that ROS and RNS responses make differential contributions to pathogenicity depending on the type of pathogen, the portal of entry to the host and the type and stage of infection.

## Conclusions

In conclusion, the exposure of fungal pathogens to host-generated ROS and RNS depends upon their route of infection and the nature and prevalence of host immune cells at these sites. Furthermore, exposure at a particular infection site changes temporally and spatially during disease progression.

Fungal pathogens differ in their sensitivity to host-generated ROS and RNS. Yet they respond by activating common mechanisms that protect them against these chemical insults. These responses, which include the detoxification of ROS and RNS, and the repair of damage caused by these toxic compounds, are generally required for normal levels of fungal pathogenicity. Some evolutionarily conserved modules contribute to the regulation of these protective responses (e.g. Hog1, Cap1 and Yap1), but there is divergence with respect to their upstream stress sensing mechanisms and some downstream transcriptional regulators (e.g. Msn2/4).

Some interesting issues remain to be addressed. Firstly, exposure to exogenous RNS is likely to lead to chemical cross-talk with endogenous ROS generated naturally by fungal cells. Therefore, the molecular output from RNS experiments probably includes the impact of these chemical interactions. This issue needs to be considered more deeply.

Secondly, fungal pathogens are simultaneously exposed to combinations of different stresses in the host, rather than to RNS or ROS alone, for example. Therefore, there is a clear need to study the impact of combinatorial stresses upon fungal pathogens. This might reveal novel molecular interactions between stress signalling pathways that are relevant to the host–pathogen interaction.

Finally, ROS and RNS signalling pathways need to be elucidated in fungal pathogens. In particular, we know little about the regulation of fungal RNS responses. Whilst recognizing that the *S. cerevisiae* paradigm has provided important insights, there is undoubtedly new circuitry, novel players and unexpected mechanisms to be discovered in fungal pathogens. Unravelling these mechanisms presents an exciting challenge for the future.

## References and recommended reading

Papers of particular interest, published within the period of review, have been highlighted as:

• of special interest
•• of outstanding interest

## Figures and Tables

**Table 1:** Regulatory proteins required for wild-type levels of oxidative and nitrosative stress resistance in pathogenic fungi.

*C. albicans*	*C. glabrata*	*C. neoformans*	*A. fumigatus*	Function	References
Signalling proteins					
Hog1^a^^,^^d^		Hog1^a^^,^^d^	SakA^a^	Stress-activated MAPK	[18,19,20^•^,21]
Ssk1^a^^,^^d^		Ssk1^a^		Response regulator	[24,25]
		Tco2^a^		Histidine kinase	[25]
Sho1^a^			Sho1^a^	Transmembrane protein	[27,28]
		Pkc1^c^		Protein kinase C	[30^•^]
Pde2^c^^,^^d^				Phosphodiesterase	[31,32]

Transcription factors					
Cap1^a^	Yap1^a^		Yap1^a^	AP-1 like factor	[33^•^,39,40]
Skn7^a^	Skn7^a^	Skn7^a^^,^^d^	Skn7^a^	Response regulator	[33^•^,60–62]
	Msn2/4^a^			Zn transcription factor	[33^•^]
Cta4^b^				Zn transcription factor	[44^••^]
		Yap4^b^		AP-1 like factor	[41^•^]
		Atf1^a^		ATF/CREB-like factor	[41^•^]

a Proteins required for resistance to ROS.

b Proteins required for resistance to RNS.

c Proteins required for resistance to both ROS and RNS.

d Proteins required for virulence.
